# Supplementary material for: Microwave photon Fock state generation by stimulated Raman adiabatic passage
Source: Nat Commun. 2017 Jan 27;8:14148. doi: 10.1038/ncomms14148 (PMC5290155; doi:10.1038/ncomms14148)
Supplement: Supplementary Information — Supplementary Figures, Supplementary Tables and Supplementary References [file ncomms14148-s1.pdf]

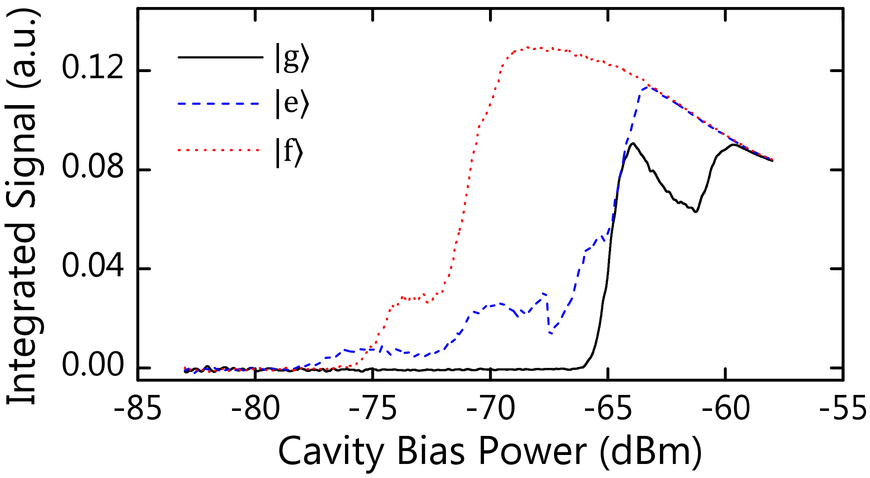

Supplementary Figure 1. **Transmon readout using state dependent cavity signal**: The state of the qubit was read out using the Jaynes-Cummings non-linear high-power readout [1]. The pulsed measurement scheme employed two cavity readout pulses (reference and measurement) that were each 3 µs long and separated by 400 µs. The difference of the two integrated pulses was averaged over 1000 shots. The best bias power for the readout cavity pulses was determined by probing the transmission response S_{21} of the cavity at its high power resonance frequency $\omega_{\mathrm{R}}/2\pi = 7.92220$ GHz. The various switching curves were obtained by initializing the transmon in the relevant states [|g⟩ (black solid), |e⟩ (blue dashed) and |f⟩ (red dotted)]. The x-axis power values are at the input of the cavity.

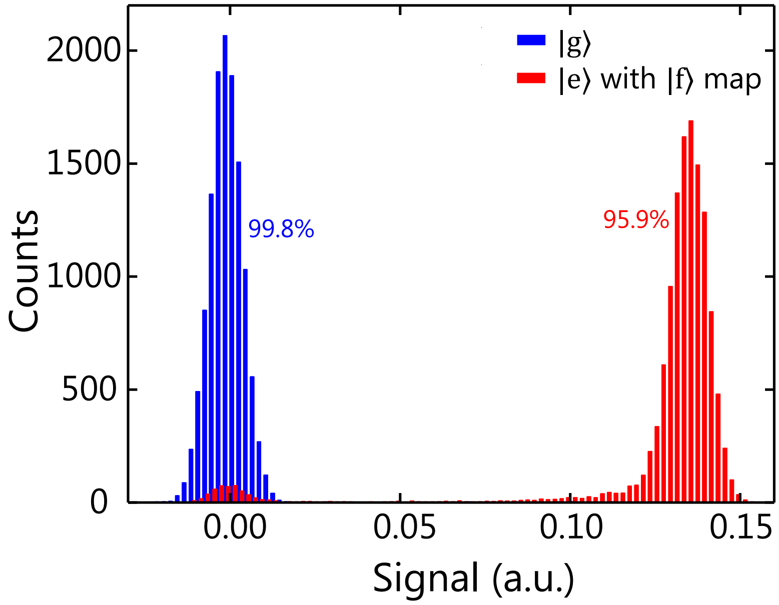

Supplementary Figure 2. **Single Shot Readout Fidelity**: The single shot readout fidelities for the ground and excited states are given when the cavity was biased at -68.5 dBm at the optimal readout point. The probability of false counts for a ground state measurement (blue) was $< 0.5\%$ and for an excited state measurement (red) was $< 5\%$. By analyzing the single-shot measurements for different cavity bias points, it was determined that the mapping to $|f\rangle$ yielded the maximum contrast and the minimum number of false counts. Hence, for Fock state generation measurements, a final mapping π-pulse was always applied to transfer any population in the $|e\rangle$ state to the $|f\rangle$ state.

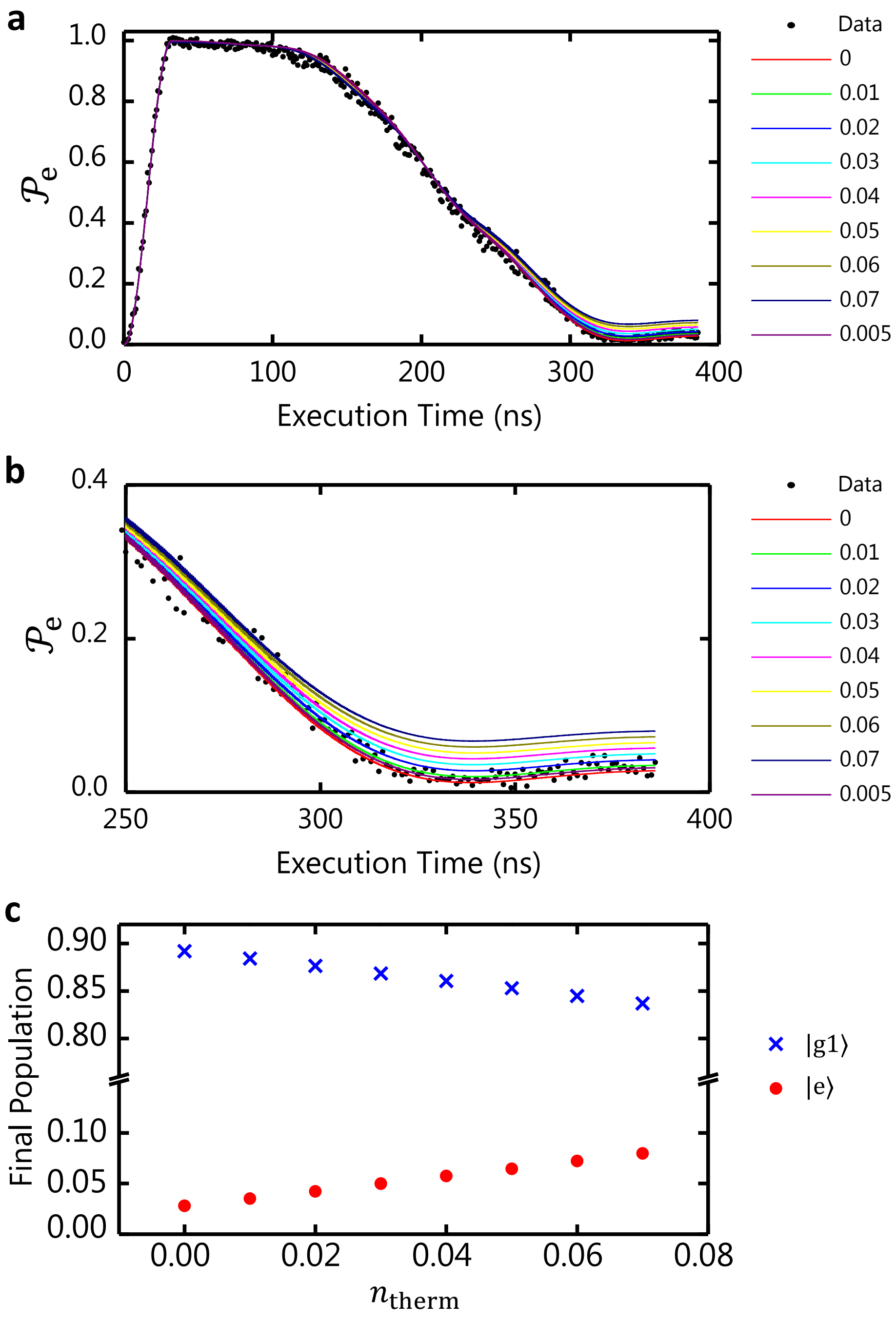

Supplementary Figure 3. **Effect of residual thermal photon population on Fock state generation fidelity**: The effect of cavity thermal photons (n_{therm}) on the process fidelity was explored through simulations with various n_{therm} in the system. Linear relationships were observed for the final residual $|e\rangle$ population and the $|g1\rangle$ population. Apart from passive cooling, no reset protocols were employed in this experiment. Any significant n_{therm} will still reside in $|g1\rangle$ after the stimulated Raman adiabatic passage (STIRAP) protocol. Any significant residual qubit excited population will reside in $|e0\rangle$ and be transferred to $|g0\rangle$ after the initialization pulse. Hence, either type of residue will not be available for the e0 $\rightarrow$ g1 transfer through STIRAP as verified by simulations. This will result in a loss of fidelity equal to the residual fraction. The residues in our system were studied by performing spectroscopy. Both peaks corresponding to $|e0\rangle \rightarrow |f0\rangle$ and $|g1\rangle \rightarrow |e1\rangle$ were found to lie within the noise floor. This gave upper bounds for the qubit and cavity excited populations of $< 4\%$ and $< 2\%$ respectively. (**a**) Full Fock state generation and (**b**) detailed version for the end of process for various n_{therm} (solid coloured lines). (**c**) Variation of final residual $|e\rangle$ populations (red circles) and final predicted $|g1\rangle$ populations (blue crosses) during generation of Fock states for different n_{therm} levels.

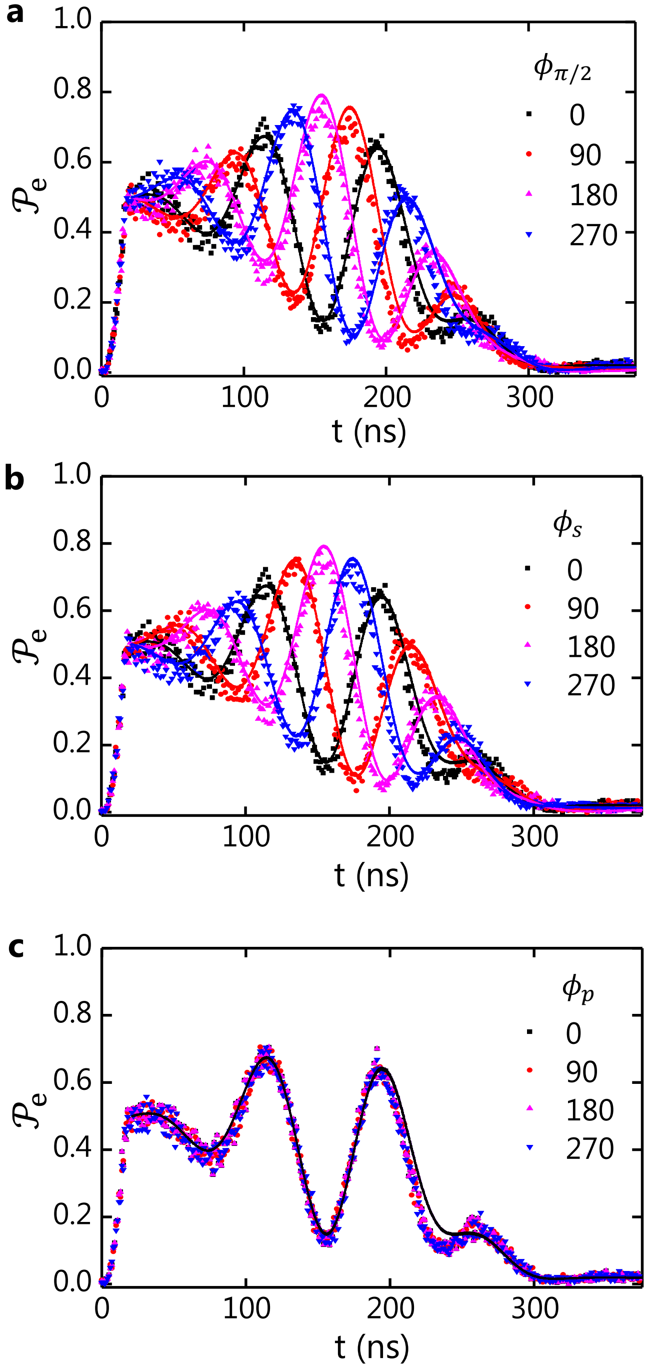

Supplementary Figure 4. **Generation of Fock state superpositions with stimulated Raman adiabatic passage (STIRAP)**: Equal superpositions between $|g0\rangle$ and $|g1\rangle$ are generated using a $\pi/2$ initialization pulse. The phase ϕ of (**a**) $\pi/2$ initialization, (**b**) Stokes and (**c**) pump pulses were changed while the other phases were held constant at zero. The experimental data points for phases 0° (black), 90° (red), 180° (magenta) and 270° (blue) are overlaid with the respective simulated evolutions of $\mathcal{P}_e$ (solid curves). The phase of the initialization and Stokes pulses exhibit complementary behavior in modifying the oscillatory features during the generation of $(|g0\rangle + |g1\rangle)/\sqrt{2}$. The phase of the pump pulse has no impact on the oscillatory features.

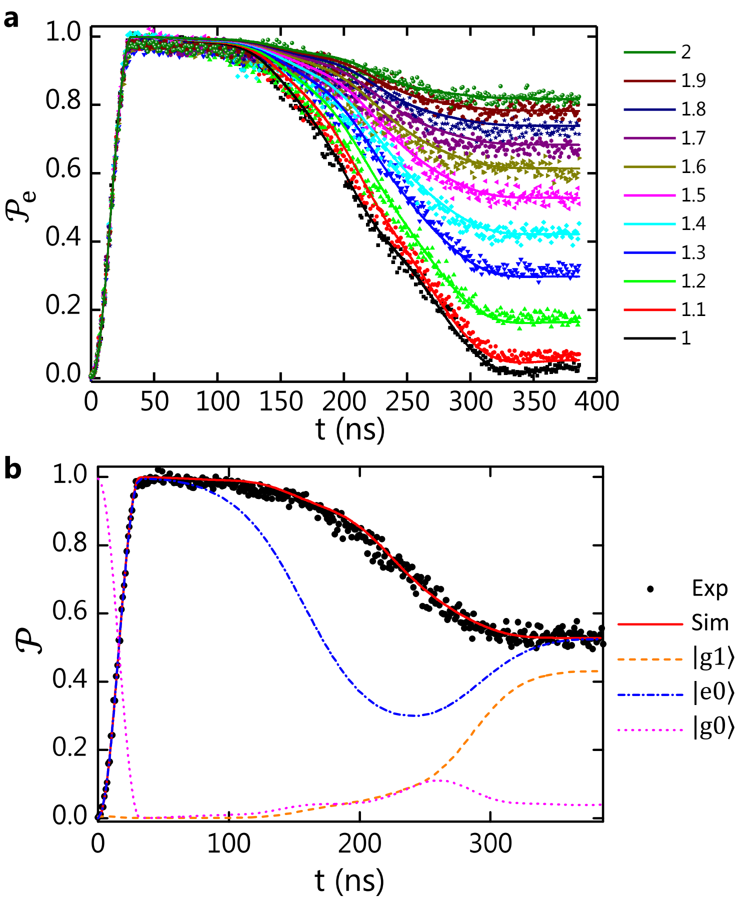

Supplementary Figure 5. **Cavity-qubit entangled state preparation**: After exciting the qubit from $|\mathrm{g0}\rangle$ to $|\mathrm{e0}\rangle$, the cavity and the qubit were entangled by performing fractional STIRAP (f-STIRAP) [2]. f-STIRAP in our system was implemented by decreasing the Stokes and pump amplitudes by the same attenuation factor β so that $\Omega_{\mathrm{s}}' = \Omega_{\mathrm{s}}/\beta$ and $\Omega_{\mathrm{p}}' = \Omega_{\mathrm{p}}/\beta$. (**a**) Plot showing probability $\mathcal{P}_{\mathrm{e}}$ of occupying qubit excited state versus time t for different Stokes and pump drive strengths by changing the drive attenuation factor β. The data points are overlaid with the pooled excited populations from simulations (solid curves) with colours corresponding to the value of β used. (**b**) Generation of the coherent superposition between $|\mathrm{g1}\rangle$ (orange dashed) and $|\mathrm{e0}\rangle$ (blue dash-dotted) when $\beta = 1.5$, with simulated contributions 0.43 and 0.52 respectively. The resultant state is approximately $0.723|\mathrm{e0}\rangle + (0.563 - 0.324i)|\mathrm{g1}\rangle$ with 6% probability for being in other states. Data points (black) are overlaid with the simulated excited population (red).

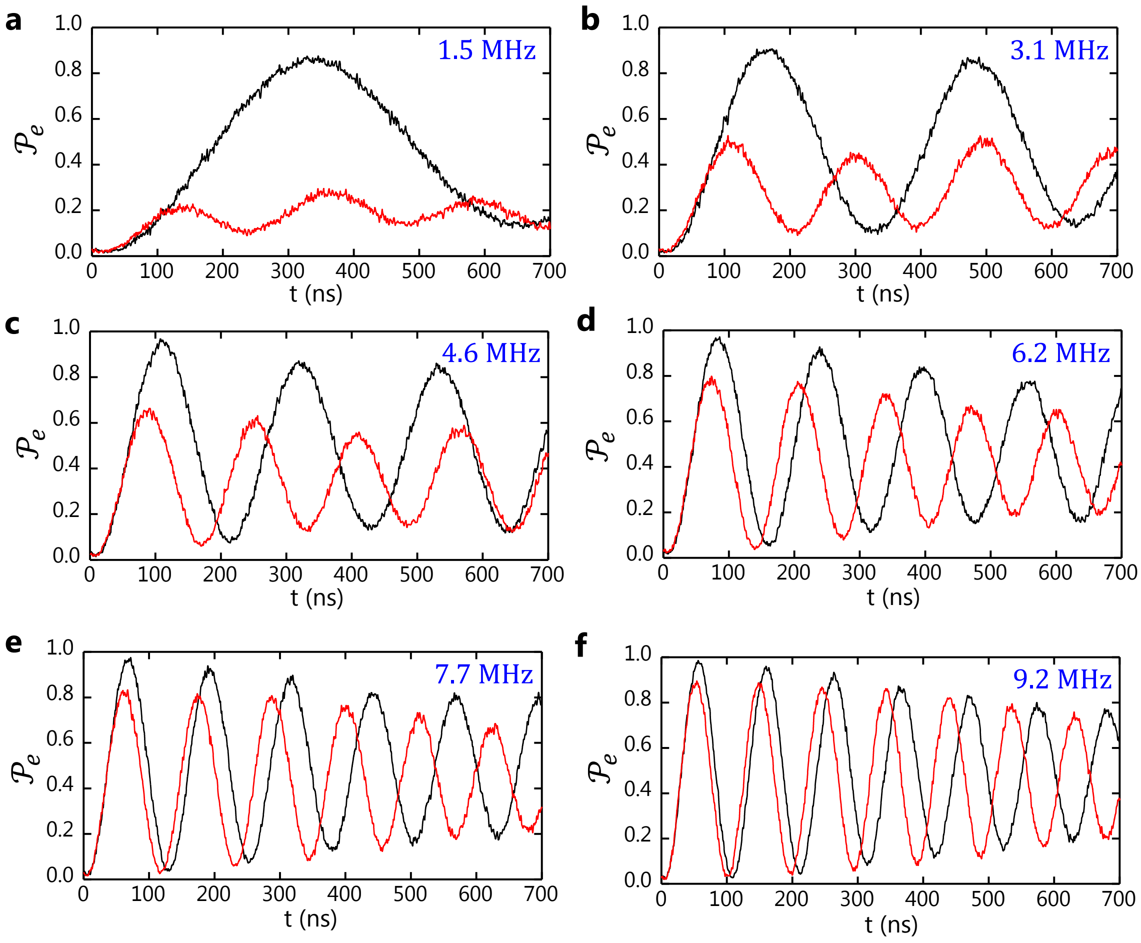

Supplementary Figure 6. **Rabi oscillations performed after $|\mathrm{g1}\rangle$ generation**: Immediately following $|\mathrm{g1}\rangle$ generation, Rabi oscillations were performed with microwave drives of frequency ω_{q} and $\omega_{\mathrm{g1}\to\mathrm{e1}}$. Different drive amplitudes were used, resulting in resonant oscillations when driven at $\omega_{\mathrm{g1}\to\mathrm{e1}}$ (black) and off-resonant oscillations when driven at ω_{q} (red) as expected. The oscillations from driving at $\omega_{\mathrm{g1}\to\mathrm{e1}}$ can be easily distinguished from higher-frequency and lower-amplitude oscillations produced by driving at ω_{q}. The nominal strengths for the Rabi drives are (**a**) 1.5 MHz (**b**) 3.1 MHz (**c**) 4.6 MHz (**d**) 6.2 MHz (**e**) 7.7 MHz and (**f**) 9.2 MHz.

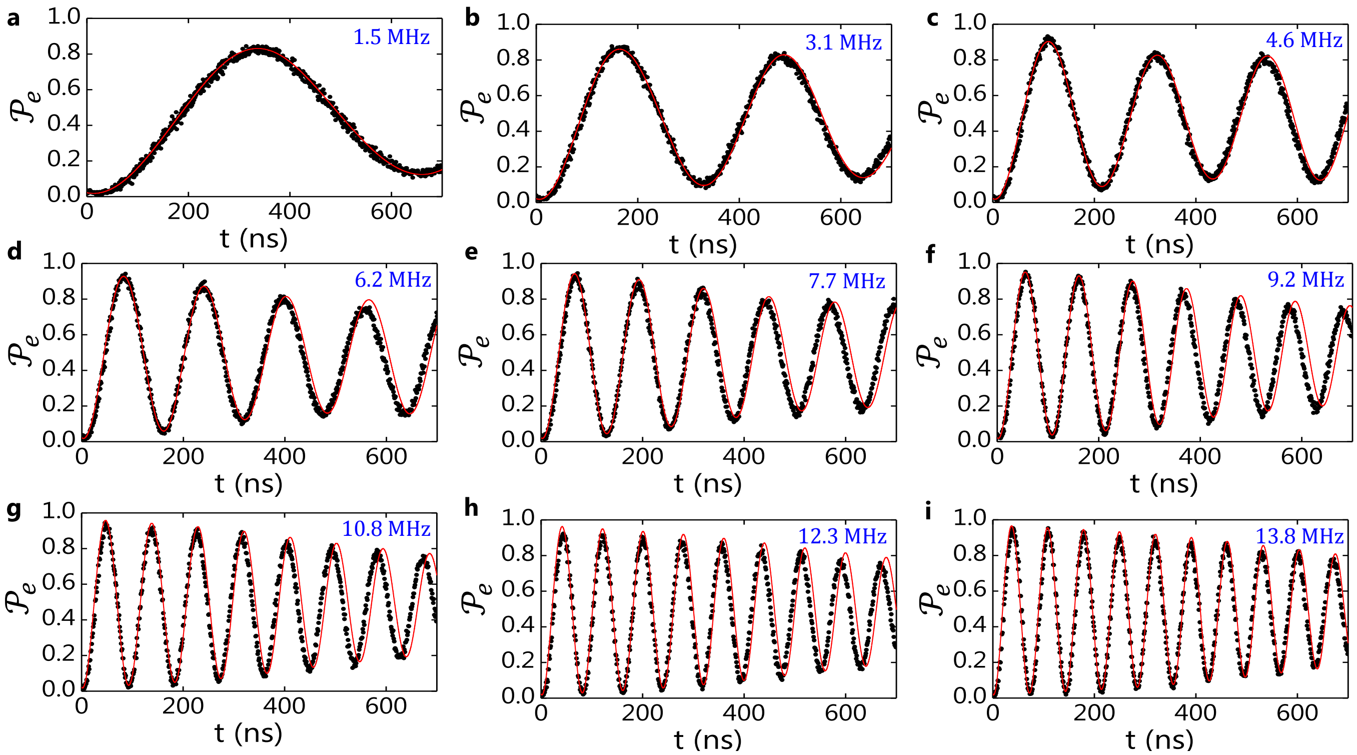

Supplementary Figure 7. **Rabi oscillations performed after $|g1\rangle$ generation under $\omega_{g1\to e1}$ drive**: Data from experiments (black points) are overlaid with simulated evolution (solid red). Examination of these curves reveals that the oscillations contain components at two different frequencies: a majority corresponding to resonant Rabi oscillations and a minority component at the off-resonant Rabi frequency. The nominal strengths for the Rabi drives are (**a**) 1.5 MHz (**b**) 3.1 MHz (**c**) 4.6 MHz (**d**) 6.2 MHz (**e**) 7.7 MHz (**f**) 9.2 MHz (**g**) 10.8 MHz (**h**) 12.3 MHz and (**i**) 13.8 MHz.

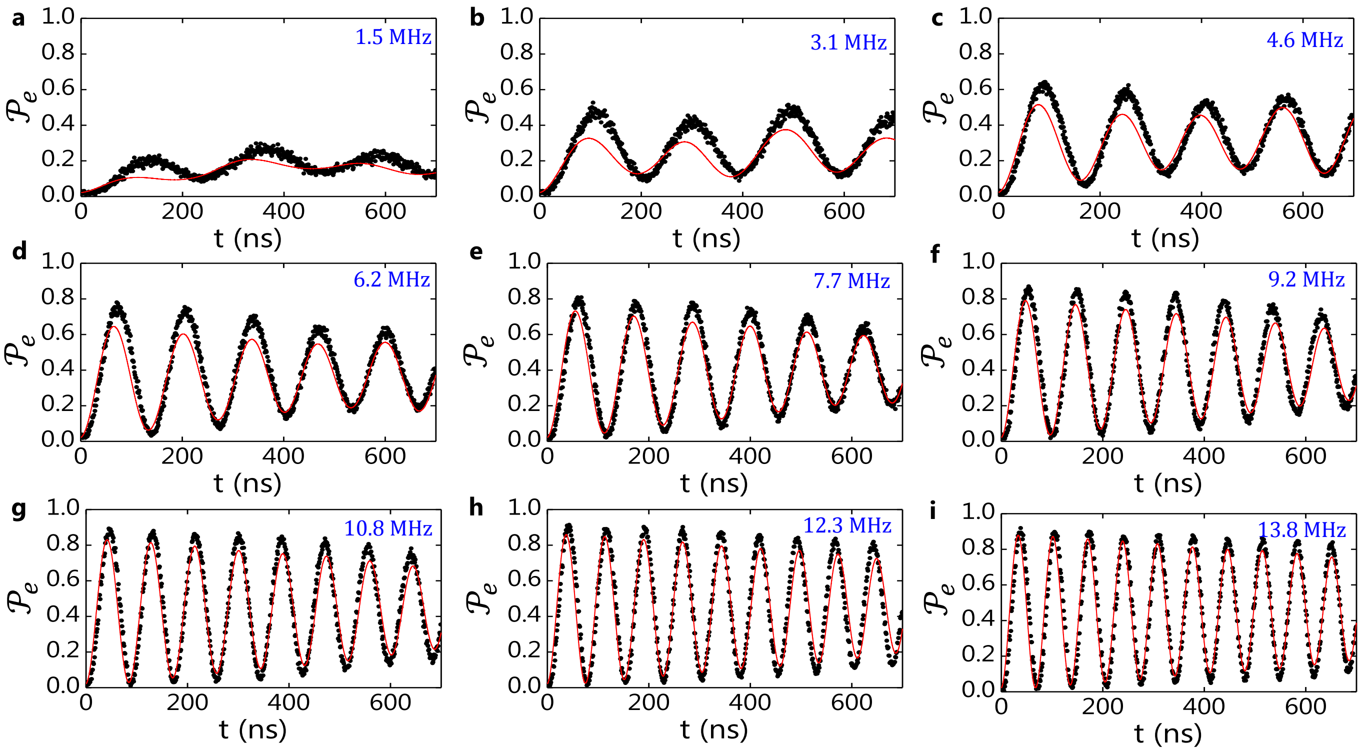

Supplementary Figure 8. **Rabi oscillations performed after $|g1\rangle$ generation under ω_q drive**: Data from experiments (black points) are overlaid with simulated evolution (solid red). Examination of these curves reveals that the oscillations contain components at two different frequencies: a majority corresponding to resonant Rabi oscillations and a minority component at the off-resonant Rabi frequency. The nominal strengths for the Rabi drives are (**a**) 1.5 MHz (**b**) 3.1 MHz (**c**) 4.6 MHz (**d**) 6.2 MHz (**e**) 7.7 MHz (**f**) 9.2 MHz (**g**) 10.8 MHz (**h**) 12.3 MHz and (**i**) 13.8 MHz.

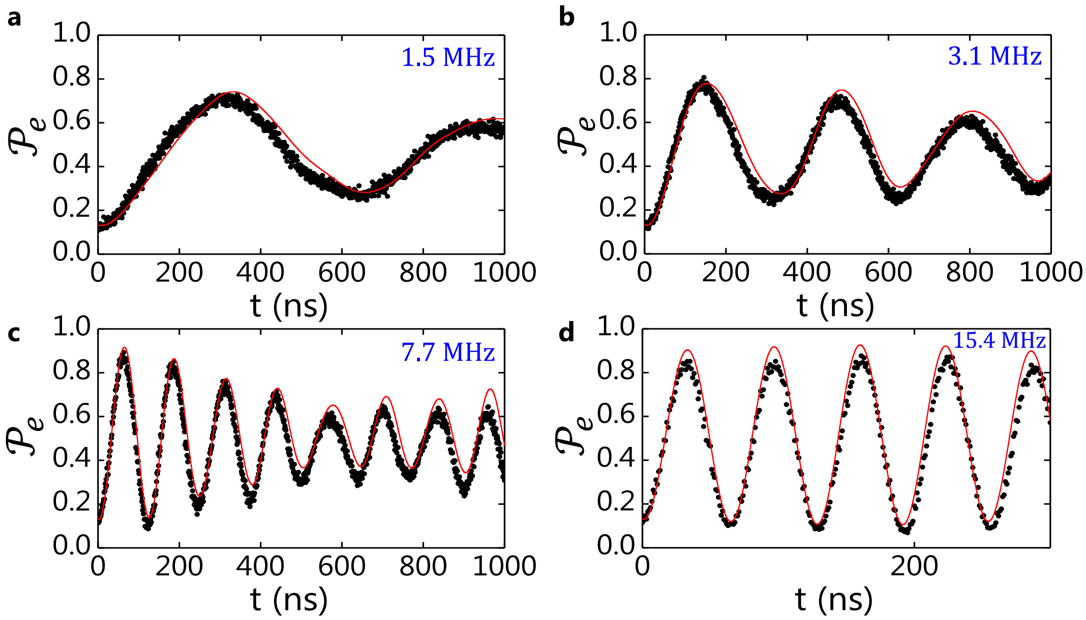

Supplementary Figure 9. **Rabi oscillations performed after $|\mathbf{g2}\rangle$ generation under $\omega_{\mathbf{g2}\to\mathbf{e2}}$ drive**: Following $|g2\rangle$ generation, a limited number of Rabi oscillations were performed at various drive strengths to verify the generated state. Data from experiments (black points) are overlaid with simulated evolution (solid red). The nominal strengths for the Rabi drives are (**a**) 1.5 MHz (**b**) 3.1 MHz (**c**) 7.7 MHz and (**d**) 15.4 MHz.

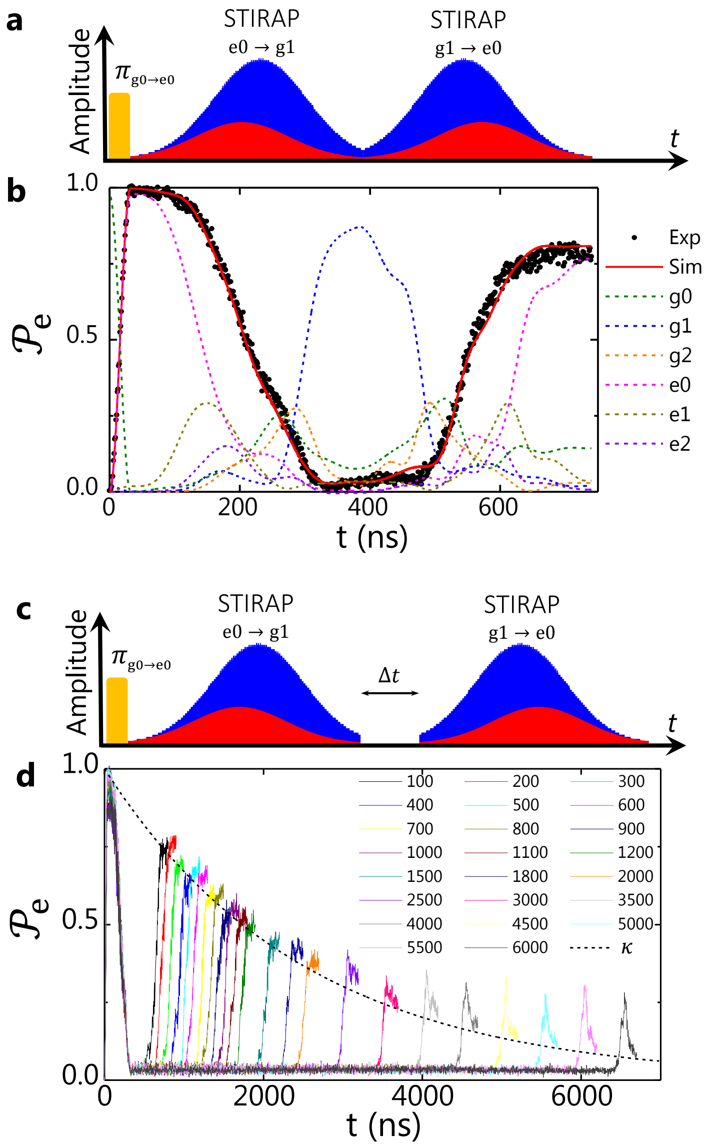

Supplementary Figure 10. **Reverse stimulated Raman adiabatic passage (STIRAP) and measurement of cavity decay rate**: As the transfer $|e0\rangle \to |g1\rangle$ was demonstrated using STIRAP, the reverse process $|g1\rangle \to |e0\rangle$ is also possible by reversing the order of pulses. (**a**) Pulse sequence to generate $|g1\rangle$ and then map back to $|e0\rangle$. (**b**) Data from reversing STIRAP (black points) are overlaid with simulated evolution (solid red). Dashed lines show simulated evolution of lowest Jaynes-Cummings levels. When performing the reversal immediately after $|g1\rangle$ Fock state generation, the recovered population is $\approx 80\%$; consistent with the significantly longer process time. (**c**) Pulse sequence to generate $|g1\rangle$ and map back with a time-delay Δt for experimental determination of the lifetime of state $|g1\rangle$. (**d**) Results of reversals performed with various Δt time-delays (solid coloured lines) along with the expected decay (κ) for the cavity (dashed black line). For longer delays, a lower population is available for the reverse mapping, and eventually ($\Delta t = 6000$ ns) almost all of the population has decayed down to $|g0\rangle$. Comparing with the naturally expected cavity decay rate $\kappa/2\pi = 64$ kHz (dashed curve) the data show excellent agreement with generation of the $|g1\rangle$ Fock state.

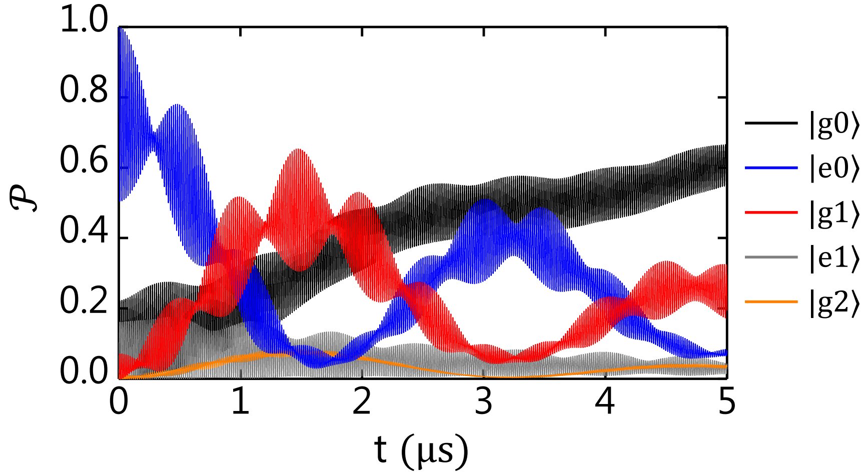

Supplementary Figure 11. **Red sideband driving for $|\mathbf{g1}\rangle$ generation**: To determine if a sideband drive technique would yield a higher fidelity, extensive density matrix simulations of the $|e0\rangle \rightarrow |g1\rangle$ sideband transition were performed. A master equation approach was used and we worked to optimize the target fidelity. We found that the maximum fidelity using sideband transitions ($\mathcal{F}_{\mathrm{g1}} \approx 65\%$) was significantly lower compared with stimulated Raman adiabatic passage. To increase the fidelity to this value we also had to increase the time of operation. Utilizing this technique also resulted in rapid oscillations and beating in the state populations. The figure shows the evolution of the populations of the five lowest levels from initial state $|e0\rangle$ in the presence of sideband drives.

Supplementary Table 1. **List of qubit and cavity parameters characterizing the system**

Parameter	Symbol	Value
Josephson Energy	E_J/h	19 GHz
Charging energy	E_C/h	211 MHz
Bare cavity frequency (high power)	$\omega_R/2\pi$	7.92220 GHz
Dressed cavity frequency (qubit in $\|g\rangle$)	$\omega_c/2\pi$	7.93700 GHz
Dressed cavity frequency (qubit in $\|e\rangle$)	$\omega_c^{(e)}/2\pi$	7.93295 GHz
Dressed qubit frequency ($\|g0\rangle \to \|e0\rangle$)	$\omega_q/2\pi$	5.52972 GHz
Mapping pulse frequency ($\|e0\rangle \to \|f0\rangle$)	$\omega_{ef}/2\pi$	5.31872 GHz
Effective cavity-qubit coupling	$g/2\pi$	69.8 MHz
Effective dispersive shift (from number splitting)	$\chi/2\pi$	–2.02 MHz
Best estimate for cavity thermal population	n_{therm}	0.005
Qubit relaxation rate	$\mathit{\Gamma}$	0.0417 µs^{-1}
Qubit dephasing rate	γ	0.0244 µs^{-1}
Cavity decay rate	κ	0.40 µs^{-1}
AWG minimum timestep	t_{min}	40 ps
Time between readout pulses	t_{RO}	400 µs
Time per readout pulse	T_{RO}	3 µs
Microwave drive coupling to the qubit	M_q	29.0 kHz/µV$_{rms}$
Microwave drive coupling to the cavity	M_c	1.10 MHz/µV$_{rms}$

Supplementary Table 2. **List of parameters used in simulating Fock state generation**

Parameter	Symbol	Value
Number of transmon levels	n_q	2
Maximum number of cavity excitations	n_{ex}	10
Non-dressed cavity frequency	$\tilde{\omega}_c/2\pi$	7.93497 GHz
Non-dressed qubit frequency	$\tilde{\omega}_q/2\pi$	5.53175 GHz
Effective cavity-qubit coupling	$g/2\pi$	69.8 MHz
$\|g0\rangle \to \|e0\rangle$ π-pulse frequency	$\omega_{g0\to e0}/2\pi$	5.5297 GHz
$\|g1\rangle \to \|e1\rangle$ π-pulse frequency	$\omega_{g1\to e1}/2\pi$	5.5257 GHz
$\|g2\rangle \to \|e2\rangle$ π-pulse frequency	$\omega_{g2\to e2}/2\pi$	5.5216 GHz
$\|g0\rangle \to \|e0\rangle$ π-pulse strength	$\mathit{\Omega}_{g0\to e0}/2\pi$	17.0 MHz
$\|g1\rangle \to \|e1\rangle$ π-pulse strength	$\mathit{\Omega}_{g1\to e1}/2\pi$	15.4 MHz
$\|g2\rangle \to \|e2\rangle$ π-pulse strength	$\mathit{\Omega}_{g2\to e2}/2\pi$	14.7 MHz
$\|g0\rangle \to \|e0\rangle$ π-pulse time	$t_{g0\to e0}$	32 ns
$\|g1\rangle \to \|e1\rangle$ π-pulse time	$t_{g1\to e1}$	34 ns
$\|g2\rangle \to \|e2\rangle$ π-pulse time	$t_{g2\to e2}$	32 ns

Supplementary Table 3. **List of parameters used in stimulated Raman adiabatic passage (STIRAP) pulses**

Parameter	Symbol	Value
Stokes drive strength (qubit-like)	$\Omega_{\mathrm{s}}/2\pi$	9.6 MHz
Pump drive strength (cavity-like)	$\Omega_{\mathrm{p}}/2\pi$	26.2 MHz
Stokes-pump pulse separation	t_{sep}	14 ns
Standard deviation of Gaussian pulses	σ	70.8 ns
Total length of each truncated Gaussian pulse	t_{len}	340 ns
Common detuning for Stokes/pump drives from level $\vert\mathrm{e1}\rangle$	δ_1	8.1 MHz
Common detuning for Stokes/pump drives from level $\vert\mathrm{e2}\rangle$	δ_2	9.7 MHz
Common detuning for Stokes/pump drives from level $\vert\mathrm{e3}\rangle$	δ_3	11.3 MHz
$\vert\mathrm{g1}\rangle \to \vert\mathrm{e1}\rangle$ Stokes Gaussian frequency	$\omega_{\mathrm{s1}}/2\pi$	5.5176 GHz
$\vert\mathrm{e0}\rangle \to \vert\mathrm{e1}\rangle$ Pump Gaussian frequency	$\omega_{\mathrm{p1}}/2\pi$	7.9248 GHz
$\vert\mathrm{g2}\rangle \to \vert\mathrm{e2}\rangle$ Stokes Gaussian frequency	$\omega_{\mathrm{s2}}/2\pi$	5.5119 GHz
$\vert\mathrm{e1}\rangle \to \vert\mathrm{e2}\rangle$ Pump Gaussian frequency	$\omega_{\mathrm{p2}}/2\pi$	7.9233 GHz
$\vert\mathrm{g3}\rangle \to \vert\mathrm{e3}\rangle$ Stokes Gaussian frequency	$\omega_{\mathrm{s3}}/2\pi$	5.5063 GHz
$\vert\mathrm{e2}\rangle \to \vert\mathrm{e3}\rangle$ Pump Gaussian frequency	$\omega_{\mathrm{p3}}/2\pi$	7.9217 GHz

Supplementary Table 4. **Sensitivity of process fidelity to stimulated Raman adiabatic passage (STIRAP) parameters**: Simulated loss of fidelity in $|\mathrm{g1}\rangle$ generation due to varying the STIRAP parameters by 10% from the predetermined optimal values.

Process Parameter	Loss of fidelity	
	Parameter Decreased 10%	Parameter Increased 10%
Stokes drive strength (Ω_{s})	3.2%	6.4%
Pump drive strength (Ω_{p})	2.9%	10.3%
Pulse truncate length (t_{len})	1.2%	3.5%
Standard deviation of pulse (σ)	0.4%	5.6%
Separation between pulses (t_{sep})	0.5%	0.5%
Common detuning from $\vert\mathrm{e1}\rangle$ (δ)	16.9%	1.8%
π-pulse length ($t_{\mathrm{g}\to\mathrm{e}}$)	2.3%	2.5%

Supplementary References

[1] Reed, M. D. *et al.* High-fidelity readout in circuit quantum electrodynamics using the Jaynes-Cummings nonlinearity. *Phys. Rev. Lett.* **105**, 173601 (2010).

[2] Vitanov, N. V., Suominen, K. A. & Shore, B. W. Creation of coherent atomic superpositions by fractional stimulated Raman adiabatic passage. *J. Phys. B: At. Mol. Opt. Phys.* **32**, 4535 (1999).
